# Host-*Toxoplasma gondii* Coadaptation Leads to Fine Tuning of the Immune Response

**DOI:** 10.3389/fimmu.2017.01080

**Published:** 2017-09-13

**Authors:** Thaís Rigueti Brasil, Celio Geraldo Freire-de-Lima, Alexandre Morrot, Andrea Cristina Vetö Arnholdt

**Affiliations:** ^1^Laboratório de Biologia do Reconhecer, Universidade Estadual do Norte Fluminense, Rio de Janeiro, Brazil; ^2^Instituto de Biofísica Carlos Chagas Filho, Universidade Federal do Rio de Janeiro, Rio de Janeiro, Brazil; ^3^Instituto de Microbiologia Paulo de Góes, Universidade Federal do Rio de Janeiro, Rio de Janeiro, Brazil; ^4^Instituto Oswaldo Cruz, Fiocruz, Rio de Janeiro, Brazil

**Keywords:** *Toxoplasma gondii*, T cells, immunomodulation, cell signalling, immunity

## Abstract

*Toxoplasma gondii* has successfully developed strategies to evade host’s immune response and reach immune privileged sites, which remains in a controlled environment inside quiescent tissue cysts. In this review, we will approach several known mechanisms used by the parasite to modulate mainly the murine immune system at its favor. In what follows, we review recent findings revealing interference of host’s cell autonomous immunity and cell signaling, gene expression, apoptosis, and production of microbicide molecules such as nitric oxide and oxygen reactive species during parasite infection. Modulation of host’s metalloproteinases of extracellular matrix is also discussed. These immune evasion strategies are determinant to parasite dissemination throughout the host taking advantage of cells from the immune system to reach brain and retina, crossing crucial hosts’ barriers.

## Introduction

*Toxoplasma gondii* is a parasite acquired through food or water contamination, followed by gut invasion and systemic dissemination. The protozoan *T. gondii* is able to escape the immune system and cross the blood–brain and blood–retina barrier reaching immune privileged sites leading to long-term infection (1). Intracellular pathogen, *T. gondii* subverts innate immunitary system interfering with host signaling pathways according to virulence based on the parasite genotype and the cell type infected (2, 3). Moreover, distinct responses can be triggered depending on inflammatory cells recruited, parasite burden, and the parasites’ molecular arrangement (4). *T. gondii* is an example of host–parasite coadaptation, and several studies have unveiled molecular interactions that allow the parasite not to exterminate the host, evading from immune responses at different levels.

## Interfering with Cell-Autonomous Immunity

Host cell gene transcription is drastically affected by *T. gondii* including those genes involved in energy metabolism, immune responses, and signaling (5–7). Initially, pattern recognition receptors such as toll-like receptors (TLRs) are able to bind parasite molecules. In mice, TLR11 and TLR12 bind to TgPRF triggering a strong IL-12 response that most effective leads to interferon gamma-inducing response genes (IRGs) (8). In humans, those genes are not functional, and TLR2, 4, 8, and 9 are effective in inducing IL-12 (9). After active invasion of host cell, *T. gondii* surrounds itself with a combination of host membrane and it is able to exclude and to recruit host proteins to the resulting parasitophorous vacuole (PV) in which it develops (3).

Rhoptry organelle initiates vacuole formation by secretion of an array of proteins that are released directly into the host cell, collectively known as RONs, forming the moving junction (MJ) (10). RONs 2/4/5/8 anchor the MJ at host cell membrane during invasion and also function as a selective sieve to host cell proteins that will be incorporated to PV (11, 12). This process assures the formation of a PV devoided of host proteins required for recruitment of endosomes and lysosomes (13).

The non-fusogenic nature of the PV is critical since it inhibits one of the cell-autonomous immunity mechanisms, the autophagy (14). Autophagosomal compartments are generated in eukaryotic cells as part of a bulk degradation system, through the formation of an initial phagophorous derived of membrane cisterna where autophagy-related proteins (ATG) are orderly accumulated leading to the fusion with lysosomal pathway (15). In non-canonical autophagy, ATG proteins can build up from preformed membranes such as the PVM, and not all ATGs are required to participate in the process (16). Extensive experimental data indicate that the autophagy machinery can promote killing of a broad variety of pathogens (17, 18) including *T. gondii*, especially in mouse models, and the IFNγ produced early in infection is crucial for that (19–23). Upon the influence of IFNγ, infected host cells respond regulating nearly 2,000 genes that are called interferon-inducible genes (24). Among those, effector molecules such as the immunity-related p47 GTPases (IRGs) and guanylate-binding proteins (GBPs) rapidly accumulate on and around the PVM, leading to the disruption of the PVM and subsequent death of the parasite in mouse cells (25). In human cells, ubiquitination and recruitment of autophagy adaptors did not require GBPs (26).

**Figure 1:**
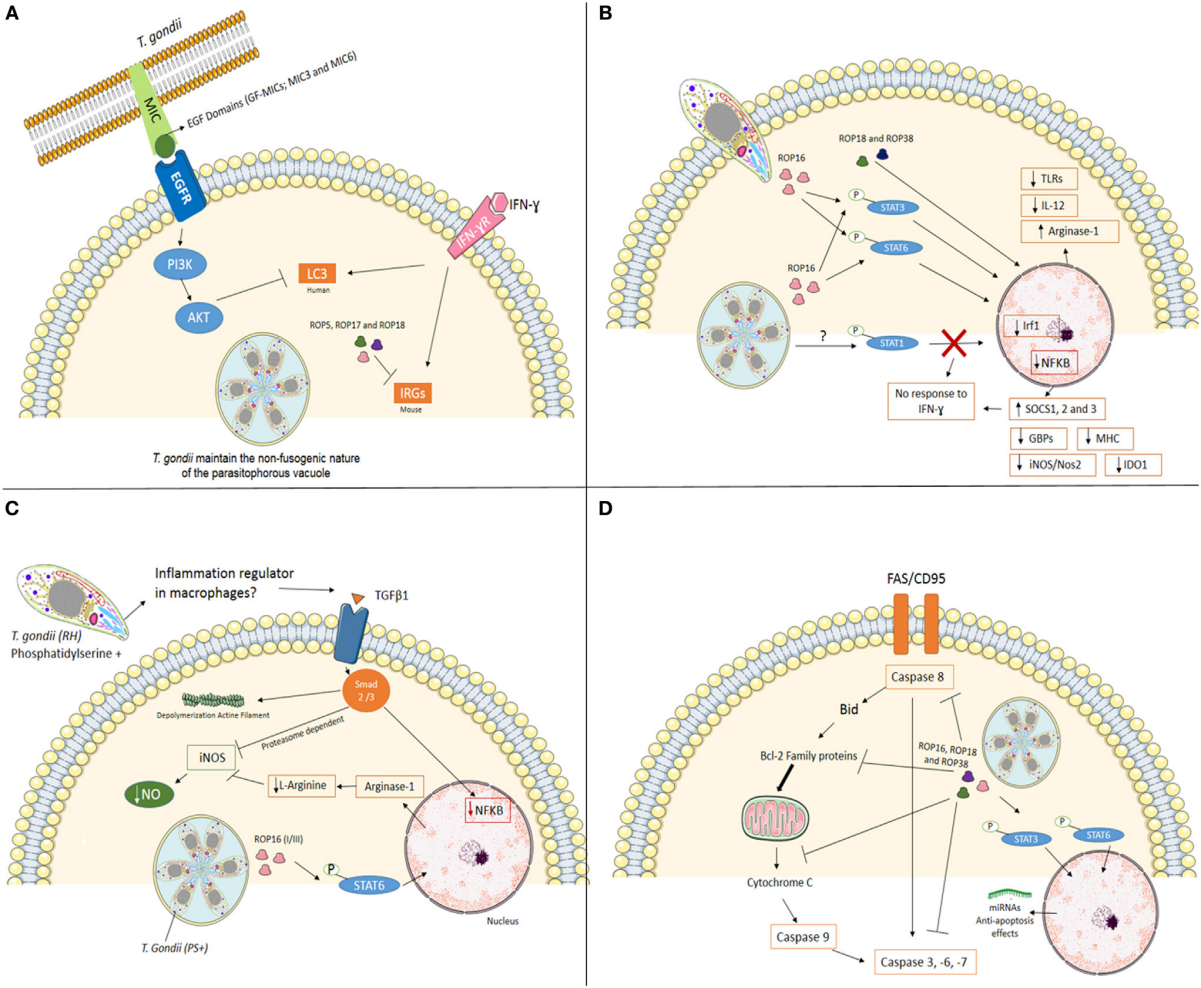
Mechanisms used by *Toxoplasma gondii*, type I strain, to modulate mainly immune system at its favor. **(A)** Interfering with cell-autonomous immunity. In infection with virulent strain (e.g., type I), the polymorphic effector proteins like ROP5, ROP17, and ROP18 cooperate to phosphorylate and inactivate mouse IRG proteins to preserve PVM integrity. In human cells (endothelial, retinal pigment epithelial, and microglia), *T. gondii* activates EGFR-Akt signaling to prevent targeting of the parasite by LC3 structures and pathogen killing dependent on autophagy proteins and lysosomal protease activity. **(B)** Cell signaling interference. The *Toxoplasma* rhoptry 16 kinase (ROP16), ROP18, and 38 can mediate the induction of arginase-1, suppress toll-like receptors (TLRs), IL-12, and nuclear factor-kappa B (NF-κB) through phosphorylation of signal transducer and activator of transcription (STAT) 3 and 6. *T. gondii* also interferes with STAT1 signaling, resulting in blockage of interferon regulatory factor 1 (Irf1), p65 guanylate-binding proteins (GBPs), inducible nitric oxide synthase (iNOS/Nos2), indoleamine 2,3-dioxygenase 1 (IDO1) and major histocompatibility complex (MHC). Furthermore, the infection upregulation SOCS1, 2 and 3. Together, *T. gondii* inhibiting pro-inflammatory response in different ways. **(C)** Silencing microbicide molecules. *T. gondii* [phosphatidylserine positive (PS+)] infection of murine blood monocyte-derived and peritoneal macrophages activated *in vitro* with IFN-γ and lipopolysaccharide (LPS) lead to a substantial decrease in NO production. Decreased mechanisms include phosphorylation of STAT6 by ROP16 resulting in arginine degradation and induction of TGFβ1 through Smad 2 and 3 leading to destruction of iNOS, actin filament (F-actin) depolymerization, and lack of NF-κB in the nucleus. **(D)** Maintaining the host cell alive. *T. gondii* has several strategies for inhibiting the initiation of the apoptotic cascade triggered by mitochondrial pathway or death receptor pathway in infected cells. The effector proteins like ROP16, ROP18, and ROP38 phosphorylate STAT3 and STAT6 and promotes mechanisms that include blocking of mitochondrial cytochrome c release, alterations of the balance between pro- and anti-apoptotic Bcl-2 proteins, degradation of caspase 8, blocking Fas/CD95-mediated apoptosis, and inactivation of effector caspases (-3, -6, -7) in infected cells.

The recruitment of IRGs and GBPs to PVM depends on autophagy-related (Atg) gene products (24, 27, 28). In mice, it has been demonstrated that Atg5 and Atg8 (LC3 in humans) are required for the proper targeting of the effectors onto the PVM of *T. gondii* (29–31). In addition, Atg12, Atg16L1, Atg3, and Atg7 are recruited to the PVM to promote parasite killing (26, 32). Infection of Atg5- and Atg3-deficient cells show decreased accumulation of immunity-related GTPase family member b10 (Irgb10) and guanylate-binding protein 2 (Gbp2) at *T. gondii* PVM (33, 34). Genotypes II and III are susceptible to the IRGs resistance system. On the other hand, infection with virulent strains (e.g., type I) has demonstrated that polymorphic *T. gondii* kinase proteins from rhoptries like ROP5, ROP17, and ROP18 phosphorylate IRG proteins in murine cells inactivating them in order to preserve PVM integrity (35–40), suggesting that in type I strain parasites can evade this cell autonomous immunity mechanism (Figure 1A).

Until recently, IFN inducible GTPases were thought to be non-functional in *T. gondii* response in human cells. Qin et al. showed that Gbp1 induced by IFNγ in mesenchymal stromal cells was responsible to decrease the number of parasites after 4 h of infection and showed that Gbp1 was found in association with at least 10% of PV (37). On the other hand, Johnston et al. (38) using A549 human epithelial cells showed that in the absence of Gbp1 parasite numbers increase rapidly. However, no Gbp1 was seen in association with PV at any moment. Clearly that are gaps in our knowledge of IRG system in the human autonomous immunity (38). Muniz-Feliciano et al. (22) showed that *T. gondii* micronemal proteins (MICs) containing epidermal growth factor (EGF) domains (MIC3 and MIC6) appeared to promote EGF receptor activation in endothelial cells, retinal pigment epithelial cells, and microglia in humans. These findings support the concept that *T. gondii* activates EGFR-Akt signaling in the host cell to prevent targeting of the PVM by LC3 (Atg8 orthologs in humans) and pathogen killing (22).

Moreover, *T. gondii* might also be killed by autophagy in mice macrophages independently of IFNγ, in a mechanism involving CD40, member of TNF receptor superfamily, and activation of ULK1, calcium/calmodulin-dependent kinase kinase b (CaMKKβ), AMP-activated kinase, and Jun-kinase (JKN are involved) (25).

## Cell Signaling Interference

*Toxoplasma gondii* modulates several signal transduction pathways once inside host cells. At the same time that an effective immune response is generated, intracellular survival strategies are adopted by the parasite. The equilibrium host-*T. gondii* is in the best interest of both allowing the establishment of long-lasting latent infection, increasing the chances of transmission to new hosts. Several *T. gondii* effector molecules have been identified that directly interact with signal transducer and activator of transcription (STAT) pathways, which influence the transcription of both pro and anti-inflammatory molecules such as IFN-γ and major histocompatibility complex class II (MHCII) (STAT1); IL-10 (STAT3); and IL-4 (STAT6) (41–44).

Rhoptry 16 kinase (ROP16) is secreted into the host cytosol during invasion and phosphorylates STAT6 in a rapid and sustained way (45). Phosphorylation of STAT6 by ROP16 mediates induction of arginase-1, resulting in arginine degradation, depriving the parasite from one important metabolite (42). Infection of mice with ROP16 knockout parasites shows that STAT3 is also phosphorylated by this kinase, suppressing TLRs and inhibiting pro-inflammatory responses at some level (42, 46, 47). ROP16 encoded by the type I/III strains, but not type II strains, maintains STAT3/6 activation for 24 h and suppresses IL-12 production from macrophages (45, 47) (Figure 1B). A recent report by Jensen et al. showed that ROP16, type II strain, induced the sustained phosphorylation and nuclear translocation of STAT5 in host infected cells, contributing to generation of protective immunity in murine gut mucosal system (48).

*Toxoplasma gondii* has also been shown to interfere with STAT1 signaling, resulting in blockage of interferon regulatory factor 1 (Irf1), p65 GBPs, inducible nitric oxide synthase (iNOS/Nos2), indoleamine 2,3-dioxygenase 1, and MHC (49–55) (Figure 1). Schneider et al. (56) showed that STAT1 is activated during infection of bone marrow-derived murine dendritic cells (BMDCs) through tyrosine 70 (Tyr70) and serine 727 (Ser727) phosphorylation with effective nuclear translocation in a ROP16 independent way. All clonal strains tested (Type I—RH, Type II—PTG, and Type III—M774.1) showed similar results, with a less effective and sustained phosphorylation induced by M774.1 (56). Besides its nuclear translocation, tyrosine-phosphorylated STAT-1 (pYSTAT1) was unable to bind to the Irf1 gene promoter and chromatin immunoprecipitation assays showed the presence of aberrant STAT1 complexes, as earlier described by Lang et al. (57) (Figure 1B).

One of the mechanisms of IFN-γ blockage is the dephosphorylation of STAT1 by SOCS1 (suppressor of cytokine signaling phosphatase) induced by positive feedback. *T. gondii* infection has been shown to induce both downregulation of SOCS1 in human fibroblasts (58) and upregulation in murine macrophages (48, 55). Infection of mice with target deletion of SOCS3 in neutrophils and macrophages results in death, as this molecule is upregulated during infection. Furthermore, the administration of anti-IL6 and IL-12 restored mice resistance to the infection (59).

*Toxoplasma gondii* induces the expression of SOCS2 in DCs through lipoxin A4 (LXA4), an arachidonic acid (Ah) with anti-inflammatory action that stimulates Ah and LXA receptors of the host cell, resulting in decreased expression of chemokine receptor type 5 (CCR5) and IL-12 secretion (60). However, enhancing transcriptions factors can also be an evasion strategy. Dense granule proteins 6 (GRA6) interferes with nuclear factor of activated T cells 4 (NFAT4), activating it *via* calcium-modulating ligand, which might lead to increased migration of inflammatory macrophages (61).

Release of dense granule protein GRA15 by type II strains, but not the type I/III strains, into the host cell cytoplasm mediates nuclear factor-κappaB (NF-κB) activation and initiates IL-12 synthesis (48, 62). On the other hand, type I strains inhibit NF-kB pathway through ROP18 and suppresses pro-inflammatory cytokine expression, resulting in the enhanced survival of the parasites in the hosts (63) (Figure 1B).

## Silencing Microbicide Molecules

Inflammatory macrophages are able to contain dissemination of infection through microbicide molecules, such as nitric oxide (NO). However, *T. gondii* infection of murine blood monocytes and peritoneal macrophages activated *in vitro* with IFN-γ and lipopolysaccharide leads to a substantial decrease in NO production (51, 52, 54). Interestingly, pretreating *T. gondii* with annexin V, which binds to phosphatidylserine (PS) reverts NO inhibition (64). The authors demonstrated that infection induces TGFβ1 through Smad 2 and 3 leading to destruction of iNOS, actin filament (F-actin) depolymerization, and lack of NF-κB in the nucleus (64). Recently, the same group showed that degradation of iNOS is proteasome dependent (65). iNOS reduced expression is also observed in microglia infected with *T. gondii*, also involving TGFβ pathways, protecting neurons from death (54). PS positive (PS+) but not PS negative (PS−) subpopulations of *T. gondii* were capable of NO inhibition after infection of murine macrophages *in vitro*, and infection *in vivo* with PS+ subpopulations leads to high parasite burden and low inflammatory symptoms at peritoneal cavity, while low or absent infection observed when PS− parasites were used with active inflammatory response observed (66). Thus, PS expression at *T. gondii* cell surface seems to be an interesting regulator of exacerbated inflammation at the entry site (Figure 1C).

## Maintaining the Host Cell Alive

*Toxoplasma gondii-*infected cells are resistant to a series of apoptosis inducers (67), allowing intracellular survival and persistence within the host cells (68). In a recent study, He et al. (69) suggested that *T. gondii* (Type I—RH) targets transregulation factors in mouse spleen cells modulating host gene expression. The genes involved in apoptosis or anti-apoptosis were both targeted by differentially expressed miRNAs, which contributes to the fate of host apoptosis process (69). The same group revealed the *T. gondii* infection can alter the transcripts at mitochondria level that are involved in several biosynthetic and metabolic processes and also in apoptosis (70) (Figure 1D).

The initiation of the apoptotic cascades is disturbed by *T. gondii* at several key points. Blocking of mitochondrial cytochrome c release is one of the mechanisms affected (71–73). The balance of pro- and anti-apoptotic Bcl-2 proteins (71, 72, 74–76) and direct inhibition of cytochrome c-mediated activation of the caspase cascade (73) were also reported. Inhibition of caspase 8, blocking of Fas/CD95-mediated apoptosis (77–79), inactivation of caspase 3 and PARP (80), as well as abrogation of Granzyme B activity in infected cells (81) are also important in maintaining the host cell alive, in favor of parasite’s survival.

Inhibition of apoptosis is regulated also at the transcriptional level. Infection of mouse splenocytes induces activation of host’s NF-κB and the transcription of antiapoptotic genes (80). After cell invasion, cells increase levels of active serine threonine kinase/protein kinase B (Akt/PKB), exploiting PI3K in a Gi-dependent way to delay host cell apoptosis (82). Furthermore, *T. gondii* phosphorylates the pro-apoptotic Bad protein to prevent apoptosis (83). These findings suggest that during the early stages of infection *T. gondii* is able to evade induction of apoptosis remaining inside the cell allowing the spreading of infection. However, there are reports indicating that ROP18 from virulent *T. gondii* strains induces apoptosis of neurons *via* RE stress (84). On the other hand, the gp130 expressed by neuronal cells protects them through IL-6, TGFβ, and IL-27 (85).

Signal transducer and activator of transcription molecules are also exploited by *T. gondii* to prevent the apoptosis. Serine proteases, like SERPIN B3 and B4, are significantly expressed in macrophages infected by *T. gondii via* STAT6 activation. Extended parasite intracellular survival in THP-1 is gain through those enzymes that ultimately inhibit apoptosis (86). In a recent work, Cai et al. demonstrated that STAT3 mediates pro-survival by upregulating the miR-17–92 that in turn targets Bim, inhibiting apoptosis in infected macrophages (87).

## Using Cells as Trojan Horses

Inflammatory cells attracted to the primary site of infection are targets of parasites that highjack the cell in order to circulate through the body inside the host cell, in a mechanism similar to a *Trojan horse*, delivering the parasite to deep tissues and immune privileged sites, such as central nervous system and eyes (88).

Our group showed that host extracellular matrix metalloproteases (MMPs) might be involved in infected macrophage dissemination (89, 90). Murine macrophages infected *in vitro* with *T. gondii* exhibit increased membrane type-1 matrix metalloproteinase (MT1-MMP) and disintegrin and metalloproteinase domain-containing protein 10 (ADAM10) while decreased levels of CD44 are observed at cell surface. On the other hand, augmented active MMP-9 is present at cell supernatant (89) resembling metastasis mechanisms used by invasive tumors (89). Upregulation of MMP-9 and -2 *via* an Erk1/2/NF-κB pathway was also observed in murine mast cells infected with *T. gondii* (91) and human macrophages infected with *T. gondii* showed increased levels of MT1-MMP, with decrease in pro-MMP-2 and pro-MMP-9, maintain the migratory capacity, although decrease some the costimulatory molecules (89).

**Figure 2:**
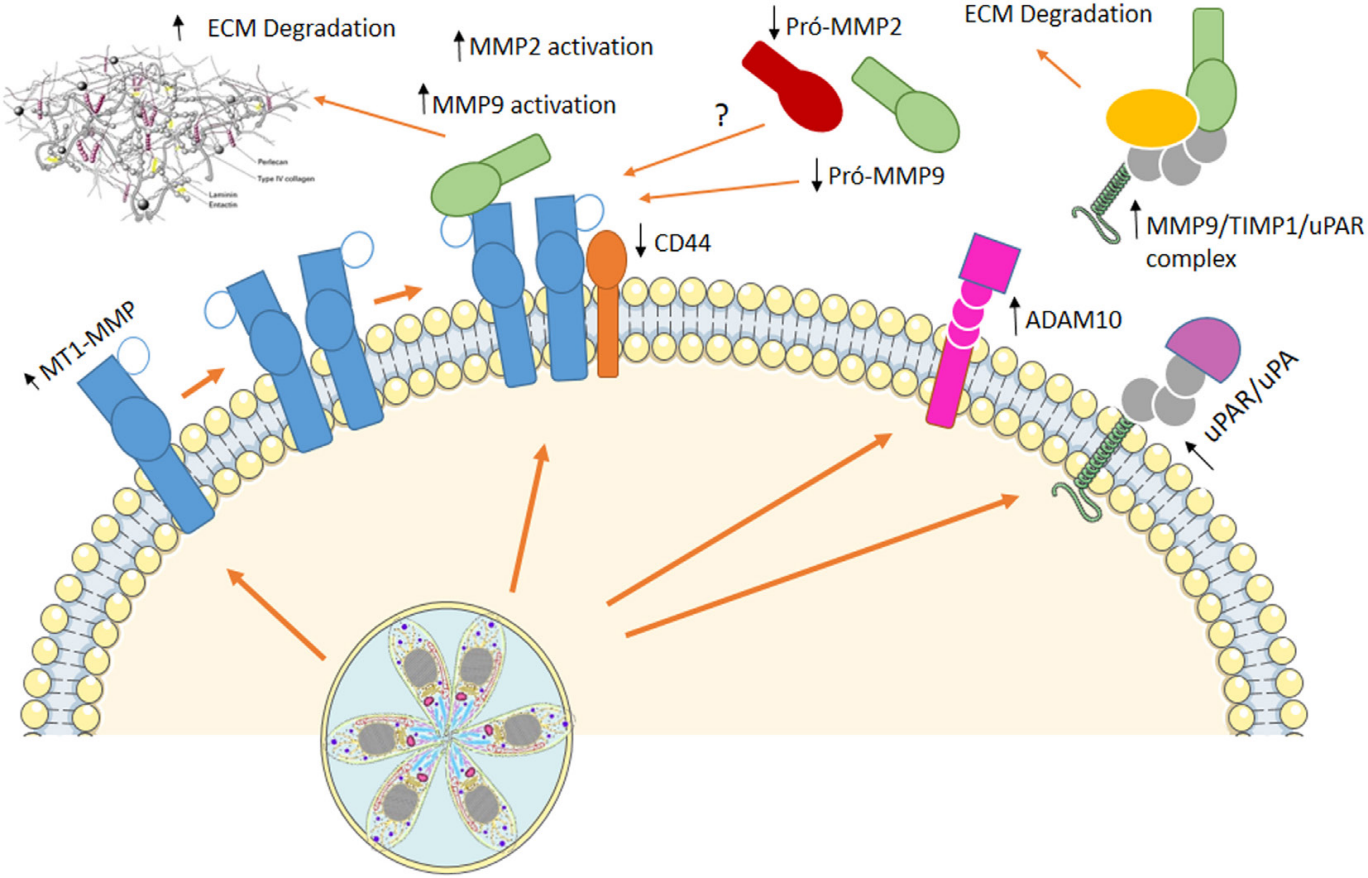
Using cells as Trojan horses. Host extracellular matrix metalloproteases (MMPs) are involved in infected macrophage dissemination. *In vitro* infection of murine macrophages induced an increase in membrane type-1 matrix metalloproteinase (MT1-MMP) and disintegrin and metalloproteinase domain-containing protein 10 (ADAM10), while decreased levels of CD44 are observed at cell surface. On the other hand, augmented active MMP-9, MMP-2, and a multiprotein complex containing MMP-9/TIMP1/urokinase-type PA receptor (uPAR) are present at cell supernatant. This mechanism resembling metastasis allows *Toxoplasma gondii* to disseminate throughout the host, reaching immune-privileged sites, where it remains in low proliferative state, with little damage to the host.

Regulation of hosts’ MMPs processing involves extra- and intracellular mechanisms upon *T. gondii* infection. Urokinase-type PA/urokinase-type PA receptor (uPA/uPAR) pathway is known to be involved with MMPs processing at extracellular space and is regulated by endogenous inhibitor of plasminogen activator inhibitor (PAI-1) and protease nexin-1 (PN-1). We demonstrated that *T. gondii*-infected macrophages secrete a multiprotein complex containing MMP-9/TIMP1/uPAR, and incubation of infected cells with PAI-1 decreases the presence of this complex at cell supernatant (90) (Figure 2).

*Toxoplasma gondii* proteinases were identified (92) and might be involved in intracellular processing of MMPs zymogens, suggesting that hosts and *T. gondii* MMPs would work in favor of parasites’ dissemination to secondary organs and to immune privileged sites. Thus, dissemination through lymphatics and leukocytes could be the main form of dissemination, and cumulative information in this subject have been gathered (88, 93).

After oral infection, *T. gondii* is found in the blood inside CD11b^+^ monocytes and inside mouse CD11c^+^ DCs at lamina propria Peyer’s patches and mesenteric lymph nodes. Infected CD11b^+^ monocytes are observed at the extravascular space in the mouse brain after 7 days of infection (94). In human infected astroglia cells, the increase of MMP-2 and MMP-9 could promote leukocyte migration during toxoplasmic encephalitis (95). MMP-2 and -9 are higher in the sera and umbilical cord of pregnant women with *T. gondii* infection (96), suggesting that MMPs might be involved in the crossing of *T. gondii* through the placental barrier. Oral infection with *T. gondii* provokes small intestine inflammation as a result of Th1 responses, that depending of mice strain and/or parasite genotype is rapidly contained (3). Muñoz et al. (97) demonstrated that MMP-2 is involved in the development of *T. gondii*-induced immunopathology. The same paper shows that this gelatinase is regulated by IL-23 *via* IL-22 but independent of IL-17 (97).

T cells from CD4^+^ and CD8^+^ lineages are essential to control bradyzoites containing cysts at the brain, T cells expressing MMP-10 are present at the brain after 21 days of infection, while T cells expressing MMP-8 are observed at 28th day of infection. At this time, astrocytes express TIMP-1, probably in an attempt to control damage (98). The presence of TIMP-1 at the brain during chronicle stages of toxoplasmosis could control degradation/activation of cytokines, decreasing exacerbated inflammatory response at the brain.

## Conclusion

Several effector molecules and mechanisms were presented here, which allow *T. gondii* to leave inside host, with little destructive effects to immunocompetent individuals. Both parasite and host have developed several strategies to decrease collateral damaging immediately after infection such as interfering with cell-autonomous immunity and cell signaling and also blocking apoptosis allowing infected host cell to remain alive. Also, controlling dissemination of parasites through metastatic-simile mechanisms, using host cell MMPs and migration, allows parasite to spread to immune-privileged sites, where it remains in low proliferative state, with little damage to the host. In order to successfully reach this semiequilibrium state between parasite–host, the initial events occurring at the parasite entrance site are crucial. Damaging control of ileitis by regulating levels of IFNγ, IL-23, and IL-17 and maintaining the fine tuning of MMPs and other enzymes and pro-zymogen enhancers, inducers, and/or converters are fundamental.

## Author Contributions

All author contributed equally for the manuscript.

## Conflict of Interest Statement

The authors declare that the research was conducted in the absence of any commercial or financial relationships that could be construed as a potential conflict of interest.
